# Investigation of hospital-acquired infections prevalence and analysis of influencing factors: a case study of a specialized infectious disease hospital in Chongqing, 2017–2023

**DOI:** 10.3389/fpubh.2024.1417645

**Published:** 2024-10-17

**Authors:** Bing Deng, Peilin Li, Yalan Liu, Juan Xie, Yaling Huang, Qingyun Sun, Shifang Su, Wenwen Deng

**Affiliations:** Chongqing Public Health Medical Center, Chongqing, China

**Keywords:** hospital-acquired infections, prevalence rate, specialized infectious disease hospital, influencing factors, infection control measures

## Abstract

**Objective:**

This study aimed to investigate the prevalence of hospital-acquired infections (HAIs) and their main influencing factors in a specialized infectious disease hospital in Chongqing from 2017 to 2023, providing reference for the formulation of precise infection control measures.

**Methods:**

A retrospective cross-sectional survey method was employed, combining bedside investigations with medical record reviews. Surveys were conducted on all hospitalized patients on a certain day of the last week of October each year from 2017 to 2023. Data collected included patients’ basic information, diagnosis, and hospital infection status. Statistical analysis, including retrospective case–control and multivariable logistic regression analysis, was performed to identify the risk factors for hospital infections.

**Results:**

The investigation compliance rate for the prevalence of HAIs in the specialized infectious disease hospital in Chongqing from 2017 to 2023 was greater than 96% each year. The prevalence rate of HAIs ranged from 0.89 to 2.52%. Hospital infection departments were mainly concentrated in general internal medicine, tuberculosis, and HIV/AIDS departments, accounting for 31.25, 26.25, and 23.75%, respectively. The most common infection site was the lower respiratory tract (54.22%), followed by bloodstream and urinary tract infections, each accounting for 9.64%. The predominant pathogens of hospital infections were *Klebsiella pneumoniae* and fungi. The utilization rate of antimicrobial drugs ranged from 20.75 to 33.25%, primarily for monotherapy. The rate of pathogen testing for therapeutic antimicrobial drug use was 82.84%, meeting national requirements. Multivariable logistic regression analysis revealed that the use of antibiotic (OR = 7.46, 95%CI 2.54–21.89, *p* < 0.001) and the presence of cardiovascular diseases (OR = 26.69, 95%CI 6.69–106.54, *p* < 0.001) increased the risk of HAIs.

**Conclusion:**

The prevalence of HAIs in specialized infectious disease hospitals remains stable, primarily concentrated in departments such as general internal medicine, tuberculosis, and HIV/AIDS. The lower respiratory tract is the main infection site, and comorbid cardiovascular diseases and antibiotic use are risk factors for HAIs. Therefore, to reduce the risk of hospital infections, it is necessary to strengthen the daily monitoring of key departments and the care of key patients. Further implementation of precise and effective infection control measures, including rational antibiotic use, regular infection monitoring and pathogen culture is warranted.

## Introduction

Hospital-acquired infections (HAIs) pose a significant threat to patient safety and public health globally, leading to increased morbidity, mortality, and healthcare costs (1, 2). These infections, acquired during the course of healthcare delivery in hospitals or other healthcare facilities, encompass a wide range of pathogens and can affect patients across all age groups and medical specialties (3). Despite advances in infection control practices, HAIs remain a persistent challenge, underscoring the need for continued research and intervention efforts to mitigate their impact (4, 5).

In the international arena, prevalence surveys of HAIs serve as one of the core tools for hospital infection control and are widely adopted. These surveys not only provide an objective understanding of the actual situation of HAIs but also enable the monitoring of long-term trends, thereby providing a scientific basis for the development of targeted infection control measures (6–8). In China, prevalence surveys of HAIs have also become an important component of the “Quality Control Indicators for Hospital Infections” (9). In recent years, both domestic and international studies have contributed valuable insights into the prevalence, risk factors, and outcomes associated with HAIs. Research conducted in general hospital settings has identified patient-related factors, such as age, comorbidities, and invasive procedures, as well as healthcare-related factors, including antimicrobial usage and environmental conditions, as significant contributors to HAIs transmission (10). Furthermore, initiatives such as antimicrobial stewardship programs and infection control bundles have demonstrated efficacy in reducing HAIs rates and improving patient outcomes (11).

However, there remains a gap in the literature regarding the specific challenges posed by HAIs in specialized infectious disease hospitals. Unlike general hospitals, these facilities specialize in the management of infectious diseases and cater to patients with complex medical conditions and compromised immune systems. As a result, the epidemiology, risk factors, and optimal prevention strategies for HAIs may differ from those in general hospital settings. Moreover, while some studies have examined the prevalence and impact of HAIs in general hospital populations, there is limited research focused specifically on specialized infectious disease hospitals (12). This gap highlights the need for comprehensive studies to elucidate the unique dynamics of HAIs in these settings and inform tailored interventions to mitigate their burden.

Therefore, this study retrospectively collected data on the prevalence of HAIs in a specialized infectious disease hospital in Chongqing from 2017 to 2023, and analyzed the factors influencing the occurrence of HAIs. The aim is to provide a scientific basis for the development of targeted interventions for hospital infection control and further provide practical guidance for clarifying infection control measures and preventing the occurrence of hospital-acquired infection events.

## Methods

### Survey objects

A retrospective survey was conducted on all hospitalized patients admitted to a certain infectious disease specialized hospital in Chongqing from 2017 to 2023, on a fixed day, including patients discharged, transferred to other departments, or deceased between 0:00 and 24:00 on that day, excluding new admissions on the same day. Referring to the age classification standard of the World Health Organization (WHO), the survey subjects were grouped by age: 0 to 17 years old as minors, 18 to 44 years old as young adults, 45 to 59 years old as middle-aged adults, 60 to 74 years old as young older adult, and 75 years old and above as older adult. This study was approved by the Ethics in Research Committee of Chongqing Public Health Medical Center. All adult patients have provided written informed consent, while for minor patients aged 0–18 years, their legal guardian or next of kin has provided written informed consent for their participation in this study.

### Inclusion and exclusion criteria

Hospital-acquired infection cases were determined according to the “Diagnostic Criteria for Hospital Infection (Trial)” issued by the Ministry of Health (Document No. WYF [2001] No. 2). Hospital infection cases were defined as infections occurring in the hospital that were neither present nor in the incubation period upon admission, including infections acquired in the hospital and those occurring after discharge. Infections acquired outside the hospital were uniformly included as community-acquired infections. Cases of infection that had already occurred before the survey but had recovered and newly occurred cases after the survey were not counted as infection cases.

The main indicators calculation formulas are as follows: (1) Actual investigation rate = actual number of surveyed individuals/number of hospitalized patients on that day × 100%. According to national regulations, the actual investigation rate of the prevalence of hospital-acquired infections should be ≥96%. (2) Hospital infection prevalence rate = number of existing new and old hospital infection cases/actual number of surveyed individuals × 100%.

### Survey methods and procedures

The survey plan strictly followed the National Health Commission’s national prevalence survey plan for HAIs. The Hospital Infection Management Department was responsible for the implementation of the entire survey. Four survey teams were set up, with one investigator assigned for every 50 beds. Investigators were selected from the infection control leaders of various clinical departments, with 3–4 investigators forming a team responsible for 3–4 wards. The survey included bedside surveys and case investigations, and the final results were combined, with discrepancies resolved through group discussions. Bedside surveys were conducted by a clinically experienced doctor from the team, while case investigations were conducted by other team members through reviewing paper or electronic medical records and conducting survey records in the real-time hospital infection management system. The number of people to be surveyed = total number of patients in the hospital on the survey day + number of discharged patients on that day − number of newly admitted patients on that day. The staff involved in the survey received centralized training and adhered to uniform survey standards before conducting the survey.

### Survey content

The survey included basic patient information, diagnosis, infection status, antimicrobial drug use, specimen collection, site of infection, pathogens, and analysis of factors affecting HAIs.

### Statistical methods

Statistical analysis was performed using SPSS 26.0. Inter-group comparisons were conducted using the chi-square test, and trend tests were performed using the chi-square trend test. A *p*-value of less than 0.05 was considered statistically significant.

## Results

### Basic information

From 2017 to 2023, a prevalence survey of HAIs was conducted, with a total of 4,523 hospitalized patients scheduled for investigation. Among them, 4,513 hospitalized patients were actually surveyed, resulting in a cumulative actual investigation rate of 99.78%. Except for the years 2017 and 2018, the actual investigation rate was 100% each year. A total of 80 HAIs occurred over the 7 years, with an overall hospital infection prevalence rate of 1.77%. Some individual cases involved multiple sites of infection, totaling 83 infection episodes and an overall infection episode rate of 1.84%. The hospital infection prevalence rates ranged from 0.89 to 2.52% from 2017 to 2023, with no statistically significant differences observed among the prevalence rates of each year (*χ*^2^ = 5.816, *p* > 0.05), as shown in Table 1.

**Table 1:** Hospital-acquired infection prevalence in a certain infectious disease specialized hospital in Chongqing, 2017–2023.

Year	Number of cases scheduled for investigation (n)	Number of cases investigated (n)	Actual investigation rate (%)	HAIs				*χ* ^2^	*P*
				Number of cases (n)	Prevalence rate (%)	Number of infection episodes (n)	Episode rate (%)		
2017	572	563	98.43	10	1.78	11	1.95	5.816	>0.05
2018	627	626	100	13	2.08	14	2.24		
2019	755	755	100	11	1.46	11	1.46		
2020	674	674	100	6	0.89	6	0.89		
2021	764	764	100	14	1.83	15	1.96		
2022	477	477	100	12	2.52	12	2.52		
2023	654	654	100	14	2.14	14	2.14		
total	4,523	4,514	99.8	80	1.77	83	1.84		

*p-value < 0.05 was considered significant.*

### Distribution of HAIs

In terms of age distribution, from 2017 to 2023, HAIs occurred as follows: 1 case (1.25%) in the age group of 0–17 years, 14 cases (17.5%) in the age group of 18–44 years, 21 cases (26.25%) in the age group of 45–59 years, 14 cases (17.5%) in the age group of 60–74 years, and 21 cases (26.25%) in the age group of 75 years and above. The differences among age groups were statistically significant (*χ*^2^ = 23.228, *p* < 0.05). Regarding gender distribution, the ratio of male to female hospital-acquired infection patients was 2.48:1, with no statistically significant difference observed (*χ*^2^ = 0.488, *p* > 0.05). In terms of departmental distribution, the top three departments with hospital-acquired infections from 2017 to 2023 were General Internal Medicine, Tuberculosis Department, and AIDS Department, accounting for 31.25% (25/80), 26.25% (21/80), and 23.75% (19/80) respectively. The differences were statistically significant (*χ*^2^ = 28.625, *p* < 0.05). as shown in Table 2.

**Table 2:** Distribution of HAIs in a certain infectious disease specialized hospital in Chongqing, 2017–2023.

Category	Total		Hospital-acquired infections		Non-hospital-acquired infections		*χ* ^2^	*P*
	*n*	%	*n*	%	*n*	%		
Year								
0–17	113	2.50	1	1.25	112	2.53	23.228	<0.05
18–44	1,427	31.62	14	17.5	1,413	31.87		
45–59	1,283	28.43	21	26.25	1,262	28.47		
60–74	1,193	26.43	23	28.75	1,170	26.39		
>75	497	11.01	21	26.25	476	10.74		
Gender								
Male	3,052	67.63	57	71.25	2,995	67.56	0.488	>0.05
Female	1,461	32.37	23	28.75	1,438	32.44		
Department								
General Internal Medicine	1,007	22.31	25	31.25	982	22.15	28.625	<0.05
AIDS	823	18.24	19	23.75	804	18.14		
Tuberculosis	1837	40.70	21	26.25	1816	40.96		
Surgery	689	15.27	9	11.25	680	15.34		
ICU	51	1.13	5	6.25	46	1.04		
Emergency Department	106	2.35	1	1.25	105	2.37		

*p-value < 0.05 was considered significant.*

From the perspective of infection sites, HAIs primarily occurred in the lower respiratory tract, accounting for 54.22% (45/83), followed by bloodstream and urinary tract infections, accounting for 9.64% (8/83) each. The composition ratio across years showed no statistically significant differences (*χ*^2^ = 58.301, *p* > 0.05). as shown in Table 3.

**Table 3:** Distribution of HAI Sites in a certain infectious disease specialized hospital in Chongqing, 2017-2023[*n* (%)].

Infection sites	Total	2017	2018	2019	2020	2021	2022	2023
Lower respiratory tract	45(54.22)	6(54.55)	8(57.14)	5(45.45)	2(33.33)	12(80.00)	5(41.67)	7(50.00)
Blood	8(9.64)	0(0.00)	0(0.00)	2(18.18)	1(16.67)	0(0.00)	4(33.33)	1(7.14)
Urinary tract	8(9.64)	2(18.18)	2(14.29)	1(9.09)	0(0.00)	0(0.00)	1(8.33)	2(14.29)
The other sites	7(8.43)	1(9.09)	1(7.14)	2(18.18)	2(33.33)	0(0.00)	0(0.00)	1(7.14)
Upper respiratory tract	4(4.82)	1(9.09)	1(7.14)	1(9.09)	0(0.00)	1(6.67)	0(0.00)	0(0.00)
Surgical site	3(3.61)	0(0.00)	1(7.14)	0(0.00)	0(0.00)	0(0.00)	1(8.33)	1(7.14)
Intravascular catheter-related	3(3.61)	1(9.09)	0(0.00)	0(0.00)	0(0.00)	1(6.67)	1(8.33)	0(0.00)
Skin and soft tissues	2(2.41)	0(0.00)	0(0.00)	0(0.00)	1(16.67)	0(0.00)	0(0.00)	1(7.14)
Deep incision	1(1.20)	0(0.00)	1(7.14)	0(0.00)	0(0.00)	0(0.00)	0(0.00)	0(0.00)
Intraabdominal cavity	1(1.20)	0(0.00)	0(0.00)	0(0.00)	0(0.00)	1(6.67)	0(0.00)	0(0.00)
Gastrointestinal tract	1(1.20)	0(0.00)	0(0.00)	0(0.00)	0(0.00)	0(0.00)	0(0.00)	1(7.14)
*χ* ^2^	58.301							
*P*	>0 0.05							

### Detection of pathogenic microorganisms in HAIs

Among the 80 cases of HAIs, a total of 36 strains of pathogenic microorganisms were cultured. *Klebsiella pneumoniae* and fungi accounted for the highest proportion, each accounting for 25% (9/36). The main detected fungi were *Candida albicans*, Aspergillus and *Stenotrophomonas maltophilia* followed by *Pseudomonas aeruginosa* and *Staphylococcus aureus*, accounting for 13.89% (5/36) and 11.11% (4/36) respectively. The differences among years were not statistically significant (*χ*^2^ = 95.158, *p* > 0.05). as shown in Table 4.

**Table 4:** Detection of pathogenic microorganisms in HAIs in a certain infectious disease specialized hospital in Chongqing, 2017–2023.

Pathogen	*Klebsiella pneumoniae*	Fungi	*Pseudomonas aeruginosa*	*Staphylococcus aureus*	*Acinetobacter baumannii*	*Escherichia coli*	Other Gram-negative bacteria	*Staphylococcus epidermidis*	*Mycoplasma pneumoniae*	Total	*χ* ^2^	*P*
2017	1	2	1	0	0	0	1	0	0	5	95.158	>0 0.05
2018	2	4	2	0	0	1	0	0	0	9		
2019	0	0	0	1	0	0	0	0	0	1		
2020	0	1	0	0	0	0	0	1	0	2		
2021	1	0	1	1	2	0	0	0	0	5		
2022	3	0	0	2	0	1	0	0	0	6		
2023	2	3	1	0	1	0	1	0	1	9		
Total	9	9	5	4	3	2	2	1	1	36		
Percentage (%)	25	25	13.89	11.11	8.33	5.56	5.56	2.78	2.78	100		

### Antibiotic utilization

During the period of 2017–2023, the prevalence survey revealed that the utilization rate of antibiotics among hospitalized patients ranged from 20.75 to 33.25%. The differences in antibiotic utilization rates were statistically significant (*χ*^2^ = 768.663, *p* < 0.05). as shown in Table 5.

**Table 5:** Utilization rate of antibiotics from 2017 to 2023.

Year	Number of cases surveyed (n)	Cases using antibiotics (n)	Percentage (%)	*χ* ^2^	*P*
2017	563	182	32.32	768.663	<0.05
2018	626	176	28.12		
2019	755	251	33.25		
2020	674	201	29.82		
2021	764	237	31.02		
2022	477	99	20.75		
2023	654	176	26.91		
Total	4,513	1,322	29.29		

*p-value < 0.05 was considered significant.*

The primary purpose of antibiotic therapy was predominantly for single-agent treatment, accounting for 95.39%, as shown in Table 6. Among non-prophylactic antibiotic users (including therapeutic and therapeutic + prophylactic use), there were 1,276 cases, accounting for 96.52% of all cases. Of these, 1,068 cases underwent microbial culture testing, with a testing rate of 82.84%. The differences in testing rates among years were statistically significant (*χ*^2^ = 335.151, *p* < 0.05) as shown in Table 7.

**Table 6:** Antibiotic utilization from 2017 to 2023 [*n* (%)].

Cases using antibiotics	Total	2017	2018	2019	2020	2021	2022	2023
Purpose of use								
Therapeutic	1,261(95.39)	167(91.76)	167(94.92)	246(98.01)	193(96.02)	229(96.02)	91(91.92)	168(95.45)
Therapeutic + prophylactic	15(1.13)	5(2.75)	2(1.13)	1(0.40)	5(2.49)	1(0.42)	0(0.00)	1(0.57)
Prophylactic	46(3.48)	10(5.49)	7(3.95)	4(1.59)	3(1.49)	7(2.95)	8(8.08)	7(3.98)
Combination application of antibiotics								
One antibiotic	1,178(89.04)	165(90.66)	150(85.23)	227(90.44)	175(87.06)	219(92.41)	93(93.94)	149(84.66)
Two antibiotics	134(10.14)	15(8.24)	25(14.20)	23(9.16)	24(11.94)	17(7.17)	6(6.06)	24(13.64)
Three antibiotics	5(0.38)	1(0.55)	1(0.56)	1(0.40)	2(1.00)	0(0.00)	0(0.00)	0(0.00)
More than three antibiotics	5(0.38)	1(0.55)	0(0.00)	0(0.00)	0(0.00)	1(0.42)	0(0.00)	3(1.70)

**Table 7:** Microbial culture testing of cases used antimicrobial treatment, 2017–2023.

Year	Cases using non-prophylactic antibiotic (n)	Microbial culture testing (n)	Percentage (%)	*χ* ^2^	*P*
2017	172	129	73.26	335.151	<0.05
2018	169	127	75.15		
2019	247	207	83		
2020	198	184	92.93		
2021	230	211	90		
2022	91	64	70.33		
2023	169	146	85.21		
Total	1,276	1,068	82.84		

*p-value < 0.05 was considered significant.*

### Factors influencing HAIs

A matched case–control study was conducted from 2017 to 2023, wherein hospital-acquired infection cases and non-infection cases among hospitalized patients were matched in a 1:1 ratio based on disease category and department. Factors including gender, age, residence, education level, length of hospital stay, presence of chronic diseases, and treatment procedures (such as catheterization, surgery, radiotherapy, chemotherapy, steroid use, and antibiotic administration) were included to construct a multiple logistic regression equation. The results revealed that the use of antibiotics significantly increased the risk of HAIs (OR = 7.46, 95% CI 2.54–21.89, *p* < 0.001). Additionally, patients with cardiovascular diseases were found to have an increased risk of HAIs (OR = 26.69, 95% CI 6.69–106.54, *p* < 0.001), as shown in Table 8.

**Table 8:** Multiple logistic regression analysis of HAIs.

Variables	*B*-value	*χ* ^2^	*P*-value	OR	95% *CI*
Gender					
Female					
Male	0.76	2.12	0.15	2.14	0.77–5.93
Residence					
Urban					
Rural	0.4	1.08	0.3	1.69	0.63–4.56
Year					
75-					
0-	−1.71	0.81	0.37	0.18	0.01–7.45
18-	−0.64	0.59	0.44	0.53	0.10–2.69
45-	−0.71	0.7	0.4	0.49	0.09–2.60
60-	−1.19	1.97	0.16	0.3	0.57–1.60
Urinary catheter					
N					
Y	0.4	0.36	0.55	1.5	0.63–4.56
Blood vessel catheter					
N					
Y	−0.14	0.05	0.82	0.88	0.40–5.55
Ventilator					
N					
Y	0.98	0.7	0.4	2.67	0.27–2.79
Chemotherapy					
N					
Y	−0.29	0.04	0.84	0.75	0.05–11.75
Antibiotic					
N					
Y	2.01	13.36	<0.05	7.46	2.54–21.89
Diabetes					
N					
Y	0.96	0.02	0.88	1.1	0.32–3.85
Cardiovascular disease					
N					
Y	3.28	21.63	<0.05	26.69	6.69–106.54
Tumor					
N					
Y	0.67	0.71	0.4	1.96	0.41–9.43
Length of stay (days)					
91-					
2-	0.34	0.1	0.76	1.39	0.17–11.52
31-	−1	0.77	0.38	0.37	0.04–3.41

## Discussion

This study investigated the prevalence of HAIs at a specialized infectious disease hospital in Chongqing from 2017 to 2023. The results revealed that the actual investigation rates exceeded 99%, surpassing the national requirement (>96%) (13). The overall prevalence of HAIs was 1.77%, and there was no statistically significant difference in infection prevalence over the seven-year period. This rate was lower than the requirement set by the National Health Commission for tertiary Grade A comprehensive hospitals (≤10%) but slightly higher than the national prevalence rate of 1.64% reported in the 2022 national survey (14). Different studies have shown variations in infection prevalence rates. For instance, studies by He et al. (15) reported an infection prevalence of 4.93% in a comprehensive Grade A tertiary hospital in Chongqing, while Liu et al. (16) reported a prevalence of 1.56% in a tertiary infectious disease hospital. Furthermore, a study by Abubakar (17) investigating medical institutions overseas found infection prevalence rates ranging from 3.4 to 40.7%. These variations suggest that differences in infection rates among hospitals may be attributed to different control measures being implemented. The notably low prevalence of HAIs in this specialized hospital may be attributed to the effective infection control measures implemented. It is worth noting that the prevalence of HAIs in 2020 was the lowest in recent years. This may be associated with the hospital being designated as a designated hospital for the treatment of COVID-19 patients shortly after the outbreak of the COVID-19 pandemic. The hospital implemented targeted reinforcement measures, including the renovation of isolation wards, intensified training for medical personnel, and closed management of medical staff and patients, which further enhanced the awareness and capabilities of hospital-acquired infection prevention and control. The strict implementation of infection prevention and control measures was emphasized (18).

Over the course of seven consecutive years, the investigation revealed that HAIs were primarily concentrated in the older adult population aged 60 and above, as well as in middle-aged individuals aged 45–59. This suggests that older age groups are more susceptible to HAIs, which is consistent with findings from studies by Li et al. (19). The top three departments where HAIs occurred were internal medicine, tuberculosis, and HIV/AIDS, which differs from previous domestic reports that mainly focused on departments such as ICU and hematology (20). This disparity may be attributed to the hospital being specialized in infectious diseases, particularly in treating HIV/AIDS and tuberculosis patients who have compromised immune systems and severe conditions, making them a high-risk group for HAIs. This is consistent with the findings of Rojas et al. (21). Regarding the site of infection, more than half of the HAIs were found in the lower respiratory tract, consistent with the findings of Vincent et al. (22), Huanhuan et al. (23), Manchal et al. (24). This highlights the importance of internal medicine and infectious disease departments in hospital infection control, with lower respiratory tract infections remaining a key focus. Continuous monitoring and reinforcement of infection control measures, such as air disinfection in wards, regular ventilation, emphasis on surface cleanliness and disinfection, as well as stricter requirements for aseptic practices, hand hygiene, and antibiotic use among medical staff, are crucial for preventing lower respiratory tract infections (25).

Microbiological testing of 80 hospital-acquired infection cases in this study resulted in the isolation of only 36 strains of pathogens, with a detection rate of 45%, lower than the findings of studies by He et al. (15) (detection rate of 67.68%). According to the diagnostic criteria for HAI, all the cases screened in this study fulfilled the definition. Each case presented with clinical symptoms and related infection indicators that escalated within 48 h of admission or within 48 h of extubation. Upon reviewing the etiological examination results, no alternative source of infection was identified, leading to the final diagnosis of HAIs. Notably, in some patients, no pathogens were detected in catheter blood, peripheral venous blood, or urine samples. This could potentially be attributed to factors such as the quality of specimen collection, detection techniques, or equipment used. This suggests the need for further improvement in patient sampling and bacterial culture testing at the hospital. The predominant pathogens cultured were *Klebsiella pneumoniae* and fungi, consistent with reports by Sun Zhigui and Wen Ximao, but different from Zhu Juno’s report which highlighted *Acinetobacter baumannii* and *Pseudomonas aeruginosa* (23, 26, 27). Moreover, fungal infections were less commonly reported among infectious agents in previous studies, which may be related to the hospital’s admission of a larger number of HIV/AIDS patients. This suggests that, in addition to focusing on bacterial monitoring, attention should be paid to fungal infections in the process of preventing and controlling HAIs, as they are of significant importance.

In recent years, the hospital has maintained an antibiotic utilization rate of around 30%, showing a downward trend overall. This complies with the requirements of the former Ministry of Health’s “2013 Special Action Plan for Clinical Application of Antibacterial Drugs,” which stipulates that the antibiotic utilization rate for hospitalized patients should not exceed 60%. This rate is slightly lower than the national average in 2022 (32.78%) but higher than the data reported in relevant studies overseas (14.5–20.5%) (28, 29). The rate of microbiological testing for therapeutic use of antibiotics reached 82.84%, with a rising trend annually, far exceeding the requirements of the National Health Commission’s Hospital Management Research Institute in 2021 and the national average in 2022. This indicates a high level of mastery among hospital doctors in terms of the timing of specimen submission and indications for antibiotic use, which is attributed to the hospital’s routine multi-departmental joint campaigns on antibiotic drug management and daily supervision efforts, resulting in significant effectiveness in antibiotic use management.

The HAIs result from the complex interplay of multiple factors (12). The results of this study indicated that the risk factors for HAIs in the hospital include the use of antibiotics and the presence of cardiovascular diseases in patients. Antibiotic misuse may lead to increased microbial resistance, thereby increasing the incidence of HAIs (27). Patients with cardiovascular diseases often have weakened immune functions or are more prone to infection due to metabolic reasons, making them more susceptible to HAIs (30). This suggests that for patients with underlying diseases and those using antibiotics, strict implementation of various control measures and enhanced supervision should be carried out in daily management.

This study has certain limitations. Since it was retrospective and most of patients had been discharged, the data primarily relied on hospital medical record systems and electronic databases, conducted on-site verification was not possible, which may have affected the completeness and accuracy of the data to some extent.

In summary, using this hospital as an example, the prevalence of HAIs in a specialized infectious disease hospital has been maintained at a relatively low level with no significant change trend, indicating overall good infection control in the hospital. The study indicates that the key populations for HAIs are middle-aged and older adult individuals aged 45 and above, with internal medicine, HIV/AIDS, and tuberculosis departments being the focus of HAIs. Lower respiratory tract infections remain the primary site of HAIs, with *Klebsiella pneumoniae* and fungi being common pathogens. The study also found that the presence of comorbid cardiovascular diseases and antibiotic use are risk factors for HAIs. Therefore, targeted and effective infection prevention and control measures should be implemented to further reduce the risk of HAIs.

## Data Availability

The original contributions presented in the study are included in the article/supplementary material, further inquiries can be directed to the corresponding author.
